# Linking geochronology and source apportionment of sediments to understand land–lake transfers in the Lake Victoria Basin

**DOI:** 10.1007/s10653-026-03451-x

**Published:** 2026-09-18

**Authors:** M. J. Watts, O. S. Humphrey, L. Tuffield, C. Gowing, A. L. Marriott, C. O. Ongore, J. Isaboke, O. Osano, William H. Blake, C. M. Aura

**Affiliations:** 1 Inorganic Geochemistry, British Geological Survey, Nottingham, UK; 2 Kenya Marine Fisheries Research Institute, Kisumu, Kenya; 3 School of Environmental Sciences, University of Eldoret, Eldoret, Kenya; 4 School of Geography, Earth and Environmental Sciences, University of Plymouth, Plymouth, UK

**Keywords:** Pb-210 geochronology, Source apportionment, Geochemistry, Sedimentation, Soil erosion, Catchment, Lake Victoria

## Abstract

Soil erosion is an increasing environmental problem in the Lake Victoria Basin (LVB), resulting in the transfer of sediment, nutrients and contaminants from terrestrial landscapes to aquatic ecosystems. These land-to-lake transfers have implications for soil fertility, water quality, ecosystem functioning and food security, yet the temporal evolution and catchment origins of sediment inputs remain poorly understood. This study combines Pb-210 geochronology, sediment geochemistry and geochemical source apportionment to reconstruct historical sediment delivery to the Winam Gulf of Lake Victoria from the Nyando, Sondu-Miriu, Awach, Luanda and Kisat river catchments. Sediment-core chronologies demonstrate a substantial acceleration in sediment accumulation from the 1960s, with the most rapid increases occurring after 2000. Sedimentation rates increased approximately seven-fold in the Nyando catchment and three-fold in the Sondu-Miriu catchment between the 1960s and 2021. Temporal changes in sediment geochemistry, including phosphorus, sulphur, calcium and organic matter, indicate increasing influence of agricultural expansion, forest clearance and urban development. Using a Frequentist sediment fingerprinting approach, historical sediment contributions were linked to specific sub-catchments, revealing that recent increases in sediment delivery were associated with shifts in sediment provenance driven by land-use change in key source areas. The integration of geochronology and source apportionment provides a multi-decadal reconstruction of land-lake connectivity and identifies priority locations for intervention. From an environmental geochemistry and health perspective, accelerated sediment transfer represents the mobilisation of nutrient-rich topsoil, organic carbon and associated contaminants into aquatic systems, with potential consequences for agricultural productivity, eutrophication, fisheries, water quality and human wellbeing. The approach offers a practical framework for targeting catchment restoration and improving ecosystem resilience across the Lake Victoria Basin and other rapidly changing tropical catchments.

**Supplementary Information:**

The online version contains supplementary material available at https://doi.org/10.1007/s10653-026-03451-x.

## Introduction

Poor land management practices leading to soil erosion, acidification and loss of organic matter have consequences for soil fertility and potentially toxic element (PTE)/organic pollutant mobilisation. These pressures are particularly challenging in developing countries, where local food systems may have limited capacity to avoid contaminated or nutrient-depleted sources (Enalbes et al., 2025; Seiler & Berendonk, 2012; Sooryamol et al., 2025). In regions dependent on subsistence farming and staple crops, erosion of fine, nutrient-rich particles can compound existing risks associated with mineral deficiencies, food safety and reduced agricultural resilience (Isaboke et al., 2026; Joy et al., 2015). Land degradation also affects aquatic ecosystems through increased sedimentation and the transfer of nutrients, PTEs, microplastics and other pollutants into receiving rivers and lakes, with implications for fisheries, aquaculture and wider ecosystem services (Aura et al., 2024; Mwebesa et al., 2025; Marriott et al., 2023). These issues are especially important in sub-Saharan Africa, where intensive tillage and rapid land-use change can increase the susceptibility of soils to erosion and reduce their capacity to support food production and ecosystem services (Dessie, 2025; Humphrey et al., 2022; Tully et al., 2015).

The Lake Victoria Basin (LVB) provides a critical setting in which to examine these land–lake transfers. Lake Victoria supports approximately 42 million people as a source of food, drinking water and livelihood, whilst aquaculture has increasing potential to support food and nutritional security. However, the lake has experienced substantial ecological change over the last century as a result of fishing pressure, land-use change and environmental degradation from catchment inputs (Nyamweya et al., 2023; Nyilitya et al., 2020). In the Winam Gulf, the World Agroforestry Centre (WAC, 2006) highlighted severe land degradation, soil erosion and sediment/nutrient delivery from surrounding catchments, warning that urgent intervention was needed to avoid further erosion, flooding and ecological harm. Despite subsequent community engagement, policy initiatives and mitigation efforts, sediment delivery and land degradation remain persistent problems.

A range of interventions can reduce erosion and sediment transfer, including terracing, nature-based solutions, cover crops, intercropping and community-scale land-management practices (Blake et al., 2018a; Hellens et al., 2024; Muoni et al., 2020). However, effective targeting of such interventions requires a better understanding of where eroded material originates, how sediment is transferred through catchments, and how these transfers have changed over time. Modelling approaches at regional and catchment scales can help to identify sediment sources and target limited resources to priority locations (Humphrey et al., 2025; James et al., 2023; Khodadi et al., 2026), while plot-scale tracer studies can evaluate the effectiveness of specific land-management practices. Fallout radionuclides such as Cs-137, Pb-210 and Be-7, and more recently Pu isotopes to quantify soil redistribution and erosion processes across different timescales and spatial settings (Alewell et al., 2017; Blake et al., 1999; Hancock et al., 2014; Mabit et al., 2008). For example, Pu-based studies in Kenyan farm plots have demonstrated how community-led mitigation can reduce erosion and nutrient losses, particularly from fine particles that are most vulnerable to transport (Dowell et al., 2024; Isaboke et al., 2026).

At the lake-basin scale, sediment cores offer an efficient means of reconstructing historical land–lake transfers. Cores collected near major river inputs can provide time-resolved records of sedimentation and sediment geochemistry, allowing changes in catchment inputs to be evaluated through time. This is particularly valuable in large systems such as the LVB, where systematic spatial sampling alone may not capture historical changes in sediment delivery. Previous LVB core studies have provided important palaeolimnological, geochemical, isotopic, pigment, spectral imaging and radionuclide datasets, but many typically rely on a small numbers of cores, have highly focussed datasets or occasionally limited chronological control (Hecky et al., 2010; Nakintu & Lejju, 2016; Outa et al., 2020; Ribbe et al., 2021; Enalbes et al., 2025).

Therefore, there is a need to connect dated sediment-core records with catchment-scale source apportionment to determine not only when sedimentation rates changed, but also which sub-catchments contributed to those changes. Stenfert Kroese et al. (2020a) suggested that whole-catchment approaches can provide important insight into changing sediment source contributions. Building on this need, the present study links sediment geochronology, core geochemistry and sediment source apportionment to reconstruct historical land–lake transfers in the Winam Gulf of the LVB.

The novelty of this study lies in combining Pb-210 geochronology, sediment-core geochemistry and source apportionment modelling to reconstruct both the timing and likely catchment origins of changing sediment delivery to the Winam Gulf. Whereas previous LVB studies often examined sediment cores or catchment erosion processes separately, this approach links dated depositional records with catchment sediment-source signatures. It therefore provides a multi-decadal reconstruction of land–lake transfers and identifies the sub-catchments most strongly associated with recent increases in sedimentation.

The overall aim of this study is to determine how sedimentation rates, sediment geochemistry and catchment sediment sources have changed through time in major river inputs to the Winam Gulf, and to evaluate what these changes reveal about land–lake transfers in the Lake Victoria Basin:

1. Reconstruct historical sedimentation rates in sediment cores from major river inputs and depositional settings in the LVB using Pb-210 geochronology,
2. Evaluate temporal changes in sediment geochemistry to identify shifts in sediment composition that may reflect changing catchment inputs, land-use pressures or depositional conditions.
3. Link dated core sediment geochemistry with catchment sediment sources using geochemical source apportionment modelling to identify changing sediment contributions from sub-catchments.
4. Assess the implications of these historical sediment transfer patterns for targeting land-management, monitoring and mitigation strategies in the LVB.

## Materials and methods

### Site description and collection

**Fig. 1:**
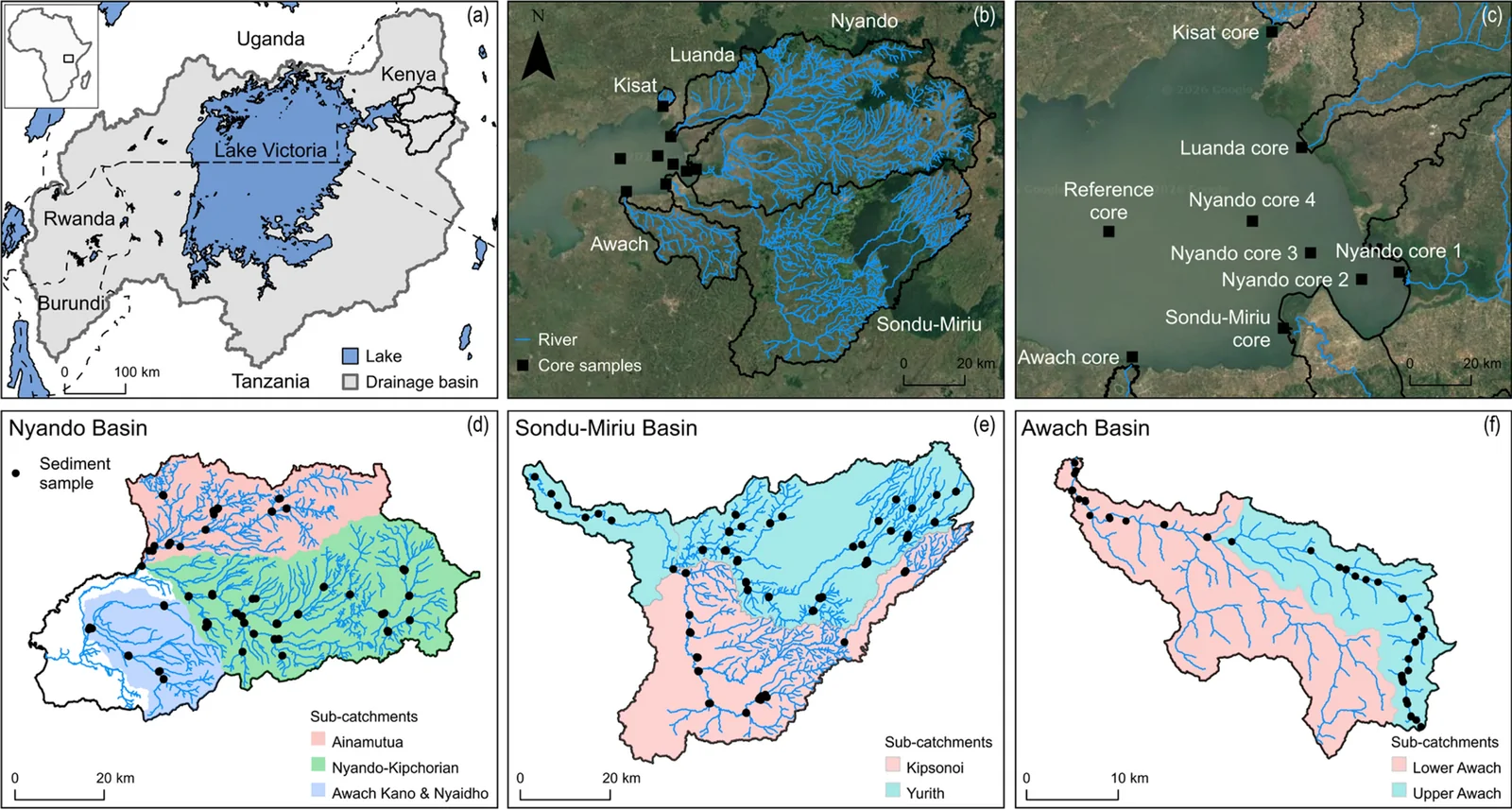
Location of the study area in the Lake Victoria Basin (**a**), all catchment in the Winam Gulf of western Kenya (**b**), the location of sediment core samples (**c**) and sediment grab locations within the three main rivers: Nyando (**d**), Sondu-Miriu (**e**) and Awach (**f**)

Sediment cores were collected across the Winam Gulf of the LVB (Fig. 1), with particular focus on the river inputs of major rivers (Nyando and Sondu-Miriu) and sedimentation plume from the Nyando catchment within the Winam Gulf previously identified by the World Agroforestry Centre (WAC, 2006), alongside additional cores from smaller river inputs to the Winam Gulf (Awach, Kisat and Luanda). The Winam Gulf as a focal point represents a significant catchment example across urban, agricultural, forestry across landscapes with elevations ranging from 1100 to ~ 2200 m. Estimates of sediment loads in the Nyando and Sondu-Miriu rivers were predicted to be 1.46 × 10^6^ Mg yr^−1^ and 5.17 × 10^5^ Mg yr^−1^, respectively (Misigo & Suzuki, 2018), from river catchments of ~ 3500 km^2^, contributing ~ 86% of measured nutrient and suspended sediment inputs into the entire LVB (Simiyu et al., 2022). The Nyando river basin contains distinct physiographic features, including the volcanic hills of the Mau Forest complex, the Nandi escarpment, the Kendu escarpment, Nyabondo plateau and the low-lying Kano plains (Owuor et al., 2012). Agriculture is the dominant land-use for large-scale tea, coffee, maize, sugar and rice cultivation (Raburu et al., 2012). The Sondu-Miriu river basin has diverse land-use types, including rainforest, managed forestry, agriculture and sub-urban settlements, light industry, hydropower generation. Conversion of rainforest to agricultural land alone reduced forest cover by 32% since the 1960s, which has significantly increased since the year 2000 (Masese et al., 2012).

The Kisat river catchment covers less than 100 km^2^ and originates in the Mangoli Forest to the east of Kisumu. The Kisat passes through densely populated slums at Obunga which lack sanitation facilities and an industrial area involving textiles, soap manufacture, fish processing, salt works, and vehicle repair shops discharging directly to the river. A subsequent sewage processing plant is no longer working, leading to multiple effluent discharge into the Winam Gulf (Kabinga et al. 2009). The Luanda River catchment is approximately 760 km^2^ originating in the Nandi escarpments at 1900 m, then passing through the lower Kano plains. The lower section is typically floodplain with a flat topography dominated by marshlands and small-scale farming (Koutsouris et al., 2010). The Awach river catchment drains into the southern coast of the Winam Gulf and is approximately 750 km^2^ originating in the Nyamira Highlands which is densely populated at 675 persons per km^2^, resulting in widespread land degradation, depleted natural forest cover and easily eroded volcanic soils. The upper Awach at an elevation of 1953 m has narrow channels, depleted forest cover with steep slopes, narrow natural riparian vegetation, high annual rainfall (1500–2000 mm) and intensive agriculture. The middle and lower reaches at an elevation of 1136 m are dominated by wide natural riparian vegetation, gentle slopes, wide channels, erratic rainfall and scattered subsistence farming (Osure et al., 2022).

Sediment samples collected from the river catchments for source apportionment analysis were selected using a nested sampling design, as described in Blake et al. (2018b). A total of 318 composite riverbed sediment samples (0–5 cm) were collected upstream of major confluences from the Nyando (117), Sondu-Miriu (120) and Awach (81) river catchments and characterised as different sources based on their geographic location in the basins. To account for natural spatial variation in riverine sediment deposition, each sediment grab sample had 8–10 subsamples spanning the width of the river channel; in accordance with best practice guidance (Collins et al., 2017; Owens et al., 2016).

### Sediment and sediment core preparation and analyses

Sediment samples were freeze dried in Kenya and ground to < 63 µm in an agate ball mill (Retsch PM400) in the UK. The organic carbon content was estimated by Loss-on-Ignition at 450 °C. Trace and major elements were determined by an Agilent 8900 ICP-MS–MS (Inductively Coupled Plasma Triple Quadrupole Mass Spectrometer) after pseudo-total digestion. Firstly, sediment samples (0.25 g) were dissolved in a mixed acid solution (HF: 2.5 ml / HNO_3_: 2 ml / H_2_O_2_: 2.5 mL) and heated to dryness on a programmable hot block with ramped heating to 180 °C. Samples were reconstituted in 50% HNO_3_, diluted to 5% HNO_3_ and further diluted for analysis in (i) collision cell mode (He-gas) for Li, Be, B, Na, Mg, Al, P, S, K, Ca, Ti, V, Cr, Mn, Fe, Co, Ni, Cu, Zn, Ga, As, Rb, Sr, Y, Zr, Nb, Mo, Ag, Cd, Sn, Sb, Cs, Ba, La, Ce, Pr, Nd, Sm, Eu, Gd, Tb, Dy, Ho, Er, Tm, Yb, Lu, Hf, Ta, W, Tl, Pb, Bi, Th and U; and (ii) H_2_-reaction cell mode for Se; and (iii) O_2_-reaction cell mode for As (at mass 91). Internal standards Sc, Ge, Rh, In, Te and Ir were employed to correct for signal drift (Watts et al., 2019). Limits of detection and both raw data and descriptive statistics for the measurement of certified reference materials (e.g. NIST CRM MESS-4, USGS CRM BCR-2) are presented for full transparency in Supplementary Table 1.

### Sediment chronology and accumulation rates

Sediment chronologies were developed from measurements of Pb-210 and 137Cs. Unsupported Pb-210 activity was determined by subtracting supported Pb-210, estimated from measured Pb-214 activity under the assumption of secular equilibrium within the U-238 decay series. Unsupported Pb-210 inventories were calculated using measured radionuclide activities and dry bulk density data for each sediment interval. Ages and dry mass sedimentation rates were then calculated following the Constant Rate of Supply (CRS) model (Appleby, 2001; Hunter et al., 2022), using cumulative unsupported Pb-210 inventories.

Dry bulk density was measured for all cores except Awach. For the Awach core, a representative dry bulk density of 0.5 g cm^^−3^ for the Winam Gulf sediments was adopted. To evaluate the sensitivity of chronology-derived parameters to this assumption, calculations were repeated using bulk density values of 0.4 and 0.6 g cm^^−3^ (± 20% of the assumed value). Because dry bulk density enters directly into calculations of unsupported Pb-210 inventory and dry mass accumulation rate, the resulting sedimentation-rate estimates varied approximately in proportion to the density assumption. This indicates that uncertainty associated with the assumed bulk density contributes an additional uncertainty of approximately ± 20% to dry mass sedimentation rate estimates for the Awach core. The age-depth relationship derived from the Pb-210 chronology is less sensitive to this assumption than calculated mass accumulation rates, but the additional uncertainty should be considered when interpreting sedimentation rates from this core and reflect only on the broader trends.

Activities of Cs-137 were measured to provide an independent chronological marker associated with maximum atmospheric fallout in 1963. However, all samples contained Cs-137 activities below the minimum detectable activity. This is common in equatorial East African sediments owing to relatively low fallout deposition and radioactive decay since peak atmospheric testing. Consequently, Cs-137 data could not be used for independent validation of the Pb-210 chronologies.

Reliable Pb-210 chronologies ideally require unsupported Pb-210 activity to decline to background levels within the recovered sediment sequence. This condition was achieved for Nyando cores 2–4 and Luanda. For Nyando cores 3 and 4, only the portion of the core containing measurable unsupported Pb-210 was used in chronology calculations. Unsupported Pb-210 activity remained above background at the base of Nyando core 1, Sondu-Miriu and Kisat, indicating incomplete inventory recovery. Chronologies for these cores should therefore be regarded as estimates of recent sediment accumulation trends rather than definitive age-depth models.

Calculated ages, dry bulk densities, unsupported Pb-210 inventories, and dry mass sedimentation rates are reported in Supplementary Tables 2–18. Uncertainties are reported as 1σ counting errors propagated through the chronology calculations, together with an uncertainty of ± 1 cm associated with sample interval thickness.

### Source apportionment modelling

To evaluate historical sediment contribution rates (1960–2020), sediment grab samples from within each catchment were statistically “unmixed” against respective sediment core samples, which were collected at the mouth of each river in the Winam Gulf and dated by Pb-210 geochronology. In this study, Frequentist sediment source apportionment modelling was conducted using the open-source R package ‘FingerPro’ (Version 2.0), available via the GitHub repository (https://github.com/eead-csic-eesa/fingerPro) (Lizaga et al., 2020b). Firstly, optimised tracers (trace and major elements) that preserve the geochemical signature of the source material during erosion and transfer to the end-point mixture were identified, as the inclusion of non-conservative and out-of-range tracers would produce erroneous models. In this study, we integrated conservativeness index (CI), consensus ranking (CR) and consistent tracer selection (CTS) methods to select the most appropriate tracers for each catchment (Latorre et al., 2021; Lizaga et al., 2020a, 2020b; Wei et al., 2026). The criteria used for selecting robust tracers were a low CTS error (< 0.05) and a high CR score (> 80). The optimised tracers were subsequently used to calculate sediment source contributions using linear variability propagation analysis (FingerPro-LVP method) developed by Latorre et al. (2025). The FingerPro-LVP method propagates source variability while avoiding the known bias associated with analysing non-linear functions, providing a more accurate approach for calculating uncertainty in unmixing models under high variability and extreme source apportionments. The FingerPro-LVP method robustly quantifies bias and mitigates mathematical errors, strengthening the reliability of sediment fingerprinting analyses (Latorre et al., 2025). Source contributions are expressed as the mean ± standard deviation of all model iterations.

### Stakeholder feedback

Co-design of the data outputs, checking of naming conventions for the catchments and sub-catchments were discussed with a range of industry, community, county and government representatives in December 2023 and June 2024 for which the broader outcomes were described in Humphrey et al., (2023, 2024). We will refer to these outcomes in the discussion.

## Results and discussion

Full datasets for each core are organised into separate geochronology and geochemistry datasets in Supplementary Tables 2–18.

### Sediment geochronology

**Fig. 2:**
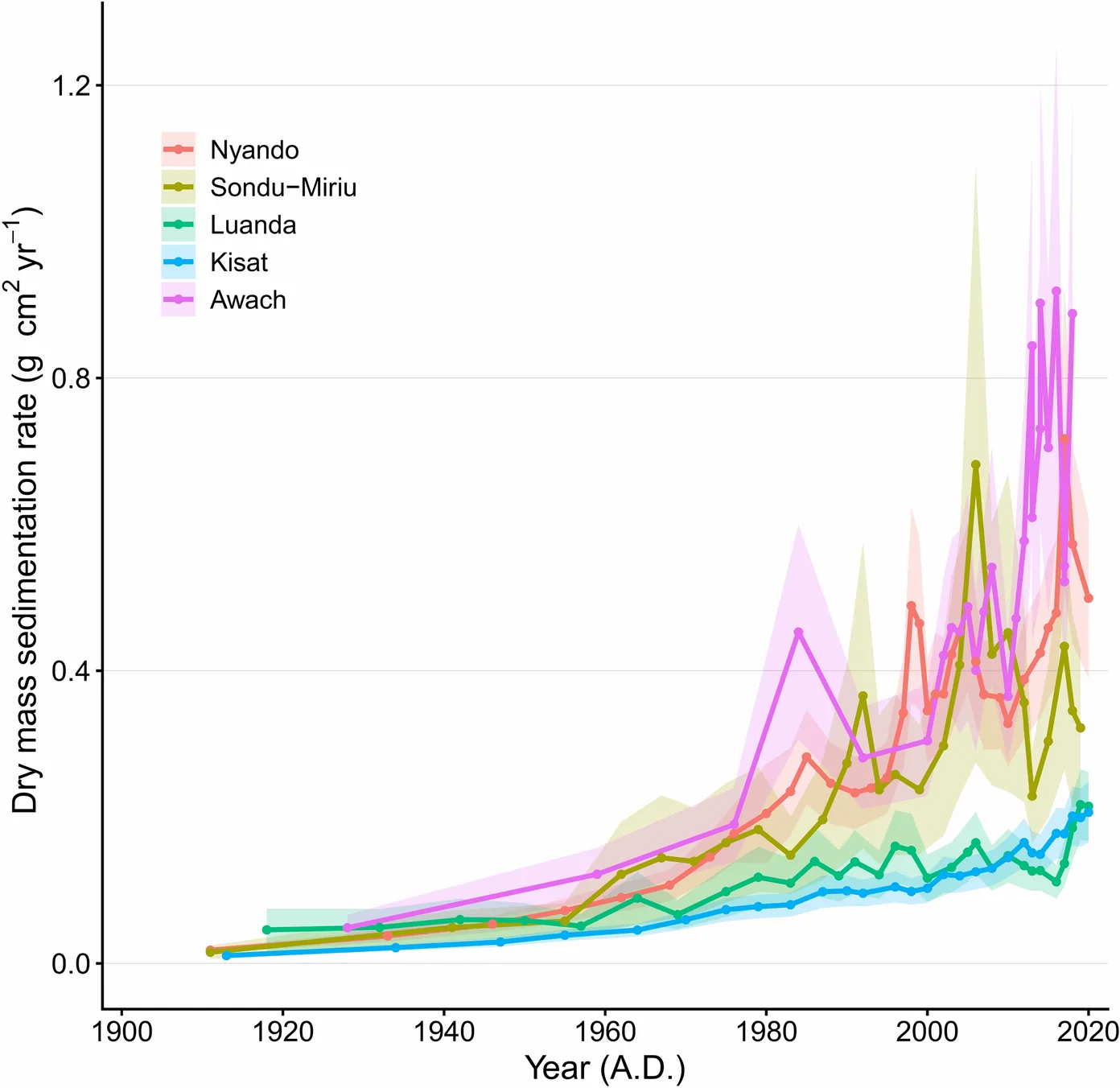
Sedimentation rates (g cm^2^ yr^−1^) at river outlets into the Lake Victoria Winam Gulf for the Nyando, Sondu-Miriu, Luanda, Kisat and Awach catchments. Errors as defined in equations 45 and 46 in Appleby, 2001

All cores exhibit a typically upward trajectory in sedimentation rates from the early 1900’s through to ~ 2020, with an inflection point in the 1960s representing a shift from colonial to independent governance focus on urbanisation in addition to clearance of land for agriculture (Njagi et al., 2022). Figure 2 provides a summary of the sedimentation rates of cores across each of the main river inputs into the Winam Gulf of the LVB. Both the Nyando and Sondu-Miriu rivers experienced the greatest increase in sedimentation rates of the rivers illustrated across this period. The Nyando had a sedimentation rate of ~ 0.1 g cm^2^ yr^−1^ in the 1960s, peaking in 2018 at 0.717 ± 0.209 g cm^2^ yr^−1^, with a rate of 0.638 ± 0.138 g cm^2^ yr^−1^ in 2021 (700% increase). The rapid increase over these years follows reported changes in land-use by Njagi et al. (2022) for the Nzoia, Sio, Yala and Nyando basins between 1985 and 2014 comprising of a 1022% increase in urban areas, 75% decrease in woodland and a 46% increase in farmland. Urbanisation land cover doubled between 1996 and 2000, whilst woodland cover decreased by 66%, which compares with the increase in sedimentation rate in this short period from ~ 0.254 to ~ 0.465 g cm^2^ yr^−1^.

For the Sondu-Mirui river, sedimentation rates increased from 0.122 ± 0.072 g cm^2^ yr^−1^ in the 1960s to 0.382 ± 0.136 g cm^2^ yr^−1^ in 2021 (300% increase). Sedimentation rates peaked in 2007 at 0.682 ± 0.072 g cm^2^ yr^−1^ coinciding with the commissioning of the first Sondu-Miriu hydroelectric power station, built between 1999 and 2007, followed by the second unit built between 2007 and 2013 (KenGen, 2026). Changes in sedimentation rate may have potential implications for the commissioning of dams or hydropower projects in similar catchments facing land-use and climate change pressures (Ochieng et al., 2022).

The smaller rivers of the Luanda and Kisat dispersing to the south and north of the Kisumu urban centre exhibited a similar inflexion point in the sedimentation rate from the 1960s to 2020, increasing 400–500% during this period, although to a lesser overall extent at 0.188 and 0.213 g cm^2^ yr^−1^, respectively. These two river catchments are significantly smaller in scale compared to the Nyando and Sondu-Miriu, although similarly originate in previously forested highland escarpment areas. Both have been subject to channelling through flatter swamp areas drained for agricultural areas developed in the pre- and post-colonial eras and via the urban centre of Kisumu from which significant run-off input will likely have contributed to sedimentation. For example, the central urban footprint of Kisumu increased from 19 to 103 km^2^ between 1969 and 2019, with broader development up to 290 km^2^ (Oloko, 2019).

The Awach river exhibited a general and slight upward trend in sediment rate from the 1920s to 2021. Comments on specific dates will need to be viewed with caution due to the use of a proxy bulk density value for this core. However, the general trend is upward, with clear peaks between 1980 and 1990 (only one point), 2000–2010 (0.3–0.5 g cm^2^ yr^−1^) and 2010–2020 (0.4–0.9 g cm^2^ yr^−1^), with both of the latter peaks supported by a greater number of data points. Hellens et al. (2024) reported an increase in soil erosion rates of 18–24% and sediment yield of 17–33% between 2018 and 2023 within the four sub-catchments of the Awach river using the Revised Universal Soil Loss Equation Model. In addition, a decline in vegetation land cover of 5–13% using Sentinel-2 NDVI satellite imagery was also reported by Hellens et al. (2024).

The reference core taken from the centre of the Winam Gulf (Supplementary Table 18) showed a gradual increase in sedimentation rate from 1940 to ~ 1996 (< 0.05–0.15 g cm^2^ yr^−1^) and then a rapid increase to 0.45 g cm^2^ yr^−1^ in 2012 and then a rapid decrease to >0.2 g cm^2^ yr^−1^ by 2016. Overall, the reference core, despite no direct deposition from a nearby river input, reflected similar trends in sedimentation rate to the cores from each of the five river inputs into the Winam Gulf—consistent upward trend and then a rapid increase in the late 1990s.

**Fig. 3:**
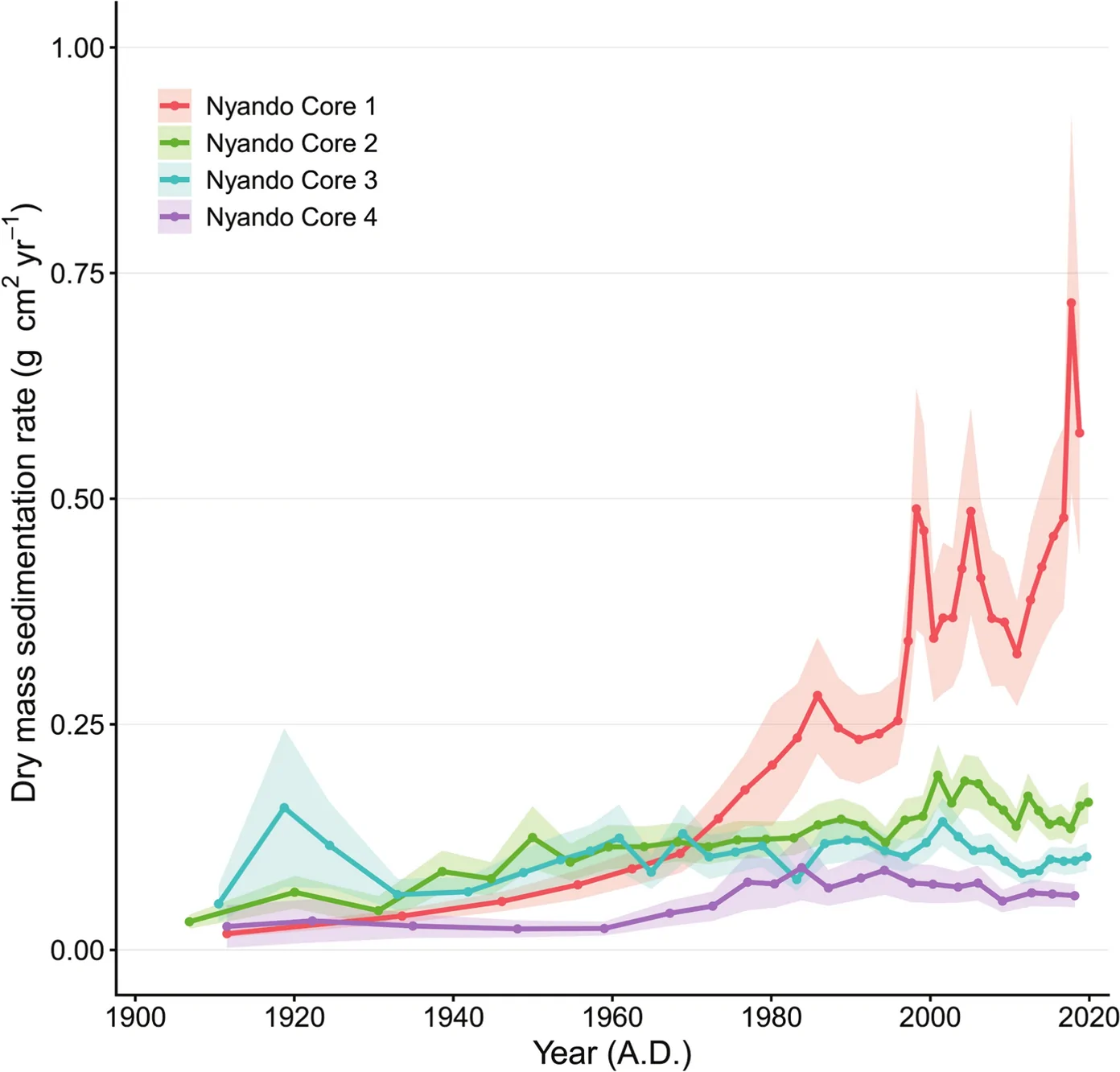
Sedimentation rate (g cm^2^ yr^−1^) from the Nyando River catchment outlet across the sediment plume in the Winam Gulf. Errors as defined in equations 45 and 46 in Appleby, 2001

The report of the World Agroforestry Centre (WAC 2006) indicated from Landsat ETM data a sediment plume of ~ 40 km^2^ across the Winam gulf from the input of the Nyando catchment. In addition to the sediment core collected from the outlet of the Nyando river (Nyando core 1), three additional cores (Nyando cores 2–4) were collected transecting the sediment plume (Fig. 1). The sedimentation rates for all Nyando river cores are presented in Fig. 3, with increasing distance from the river outlet. The Nyando core 2 provides data only back to the 1960s, but with a moderately higher sedimentation rate than the Nyando cores 3 and 4 further from the river outlet. The Nyando core 3 shows no discernible change and the Nyando core 4 an approximate two-fold change in sedimentation rate from the 1960s, albeit from a low starting point of less than 0.1 g cm^2^ yr^−1^. The plume presumably is representative of smaller particle deposition, carrying further from the river outlet, with the cores showing the majority of deposition close to the river outlet, but with a wide dispersion within the Winam Gulf and beyond into the wider LVB.

### Sediment core geochemistry

For the Nyando core 1 (plotted in Supplementary Table 3), the P, S and organic matter (%LOI) concentrations were consistent until the 1960s, after which they decreased by 20–30%, remaining consistent until the 1990s when P and S almost doubled in concentration within 10 years and then consistent to 2021 for S, but for P and organic matter, a sustained increase to 2010, after which plateauing to 2021. A distinct change in Ca occurred from 2005, increasing ~ 25% within a year and then sustained at a consistent concentration thereafter. In general, across all cores there was a noticeable increase in concentrations (peaks-noise) post-1990 for a range of elements particularly for P, S, Ca, organic matter, likely as a result of increased land clearance for agriculture and forestry. There was a lag effect for the Nyando cores 2–4, with increasing distance from the input to the Winam Gulf, post 1990 for Nyando cores 2 and 3 and post 2005–2010 for Nyando core 4. For Nyando cores 2–3 and to a lesser extent in Nyando core 4, concentrations of rare earth elements, increased substantially in Nyando cores 2–4 post 1970–2021.

The majority of cores exhibited no change in K sedimentation (potential proxy for clay) with the exception of Nyando core 3 which showed a gradual increase from 1910 to 2021, a peak between 2000–2010 for the Sondu-Miriu and downward step change from 2010 in the Luanda core. The Luanda core exhibited a broad peak (+ 50%) in S concentrations from the 1930s to 1980s, then stable until a similar peak between 2010 and 2020. Similarly, S, Fe and Ni showed constant concentrations to 1990, dropped by up to 50% and then returned to the previous level in line with the organic matter content for both the Luanda and Sondu-Miriu cores, possibly relating to the sulphide content from mineral sources or anoxic deposition. For the Sondu-Miriu core, a reduction in U, Ce and rare earth elements aligns with organic matter after 1979, indicating possible changes in sediment sources and most notable from 2005 to 2010, aligning with the commissioning of hydro-projects. However, in general across all cores, rare earth element distributions exhibited a consistent deposition trend, suggesting a largely constant mineral source of sediment, with the increase in sedimentation rate and changes in chemical composition largely driven from surface geochemistry—land clearance and soil erosion. This aligns with Stenfert Kroese et al. (2020b) who reported that catchments where land was converted to agriculture with bare soil and poor land conservation generated six times more sediment yield.

### Source apportionment

**Fig. 4:**
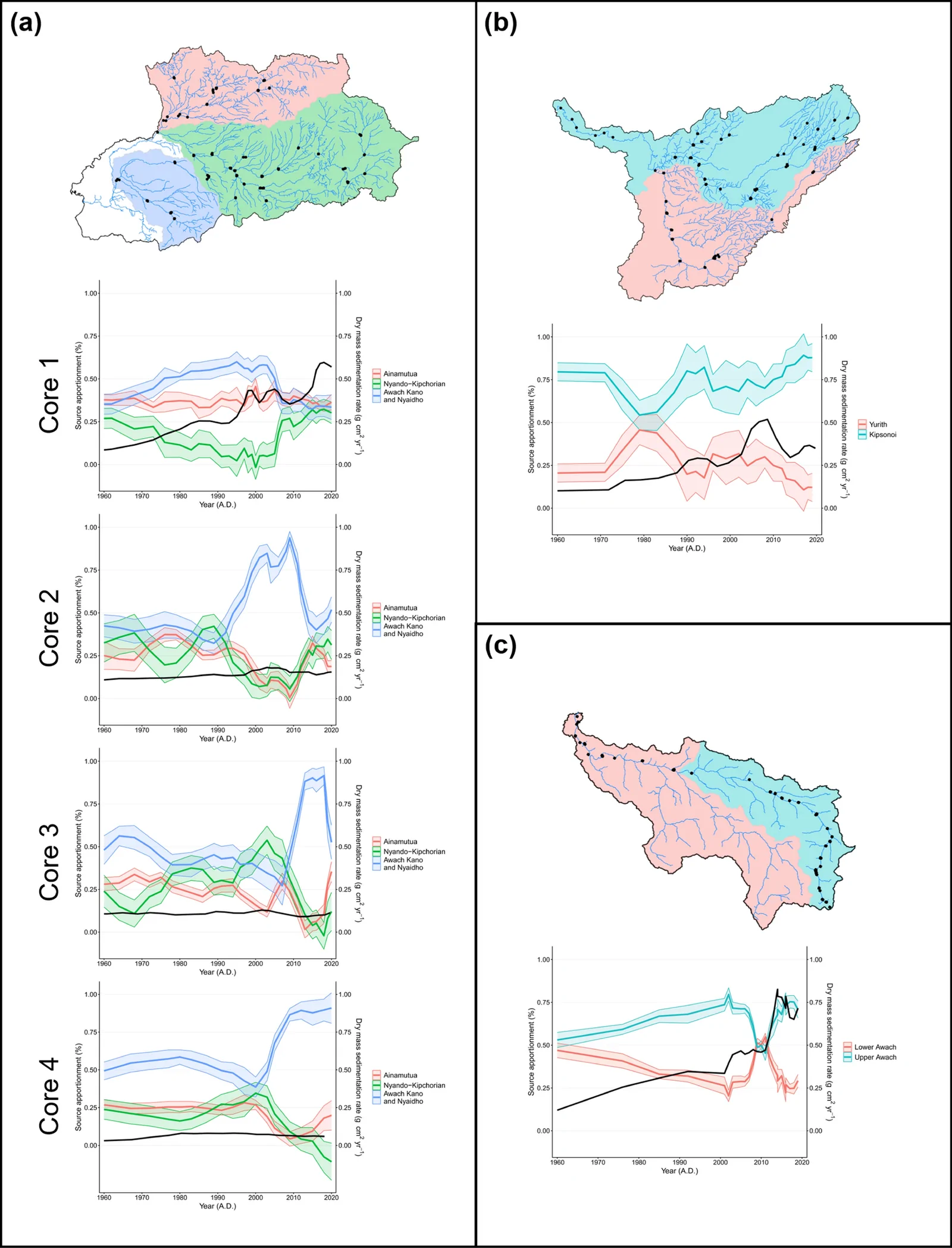
Relative sediment source contributions from the Nyando (**a**), Sondu-Miriu (**b**), and Awach (**c**) catchments to their respective sediment cores between 1960 and 2020. Catchment maps show the spatial distribution of source apportionment analysis. Time-series plots show reconstructed sediment source contributions (%; primary y-axis) together with sedimentation rates (g cm^−2^ yr^−1^; secondary y-axis). Both time series are presented as three-point moving averages

To provide insight into the origin of sediments within the catchments we deployed sediment tracing to link the geochemistry-geochronological data of the sediment cores to the catchment ‘grab’ sediment samples from the tributaries of the Nyando, Sondu-Mirui and Awach catchments. The geochemical tracer properties for the Nyando and Sondu-Miriu were reported in Humphrey et al. (2025), and the sediment samples for the Awach river are presented in Supplementary Table 17. The sediment tracing tool provided source apportionment for the origin of sediments at each time point in the sediment core to show changes in their relative contributions to the overall sediment output from individual sub-catchments. Figure 4 provides a summary of the plots for the relative sediment source contributions from the 1960s to 2020 for the outlets of the Nyando cores 1–4, Sondu-Miriu and Awach catchments—raw data is presented in Supplementary Tables 19–24.

The Nyando core 1 (Fig. 2 and Supplementary Table 19) shows a relatively stable input from each of the sub-catchments of the Nyando until a rapid increase between 2000 and 2005 in relative inputs from the Nyando-Kipchorian sub-catchment. In contrast, the relative input from the Awach Kano and Nyaidho sub-catchment, the low-lying Kano plain historically prone to erosion (WAC 2006), which had been the highest pre−2000 showed a rapid reduction in relative sediment input during this period. The Ainamutua sub-catchment, which experienced a degradation in land management as forests were cleared from pre−1960s to subsistence farming, remained steady (~ 38%) as a relative input of sediment. It is worth noting from Fig. 2, the overall sediment input to the LVB increased substantially from 1960, with further acceleration from the year 2000, hence sediment contributions from all sources will likely have increased in this period, albeit increasingly more significant from the Nyando-Kipchorian sub-catchment. Nyando cores 2–3 (Fig. 3 and Supplementary Tables 20–22) across the sediment plume transect showed a differing trend to the river outlet, with a peak in relative sediment contribution from the Awach Kano and Nyaidho sub-catchment and equivalent reduction from the Ainamutua and Nyando-Kipchorian sub-catchments. For Nyando core 2, the increase from the Awach Kano and Nyaidho sub-catchment occurred from the 1990s and returned to a balanced contribution, similar to Nyando-1 by ~ 2015. The Nyando core 3 showed a similar pattern, but delayed, starting from ~ 2008 and reducing again by 2020. For Nyando core 4, the increase in relative contribution from the Awach Kano and Nyaidho sub-catchment started ~ 2000 and showed a delayed response in returning to a similar pattern as for Nyando cores 1–3. Overall, Nyando cores 2–4 demonstrated a disruption in the source contribution from the Awach Kano and Nyaidho sub-catchment the 1990s, with a slightly delayed response along the transect and from the Nyando-Kipchorian sub-catchment for Nyando core 1. Njagi et al. (2022) reported a 75% decrease in tree cover across the Nyando catchment between 1985 and 2014, with most of this change occurring between 1996 and 2000 (66%), and similarly significant changes in urban cover of 1022% and 200% in these periods. The notable changes in observations from Njagi et al. (2022) were a likely contributor to the increase in sedimentation rates from the year 2000 but also reflected in the changes from within each sub-catchment. Specifically, the Tinderet Forest, within the Ainamutua and Nyando-Kipchorian sub-catchments, lost 26.6 km^2^ of tree cover between 2000 and 2020, equivalent to a 10% reduction in tree cover (Potapov et al., 2022), which would significantly increase soil erosion and sedimentation loads.

The Sondu-Miriu sediment core exhibited a relatively stable pattern in the source of sediments (Supplementary Table 23) compared to the Nyando core 1 from 1960, except for a mirrored rise and fall in sediment source contributions from 1970 to 1990. From 2005, a greater proportional contribution in sediment was shown from source 2 through to 2020, coinciding with the commissioning of hydro-projects, but most likely due to the increase in deforestation, with 32% lost over the last 60 years, but most notably since 2000. Similarly to the Nyando catchment, the overall sedimentation rate increased rapidly from 1960, particularly from the year 2000, albeit with the upward sedimentation rate interrupted during the same period as the relative contribution of sources were disrupted between the Kipsonoi and Yurith sub-catchments. The trending relative contribution of sediments for the sediment core from the Kipsonoi and Yurith sub-catchments for the last 20 years towards 80–90% from the Yurith sub-cathcment by 2017 aligns with the sediment collections from across the catchment in 2022 reported by Humphrey et al. (2025). The Kipsonoi sub-catchment is dominated by steep slopes and covered by herbaceous vegetation, whereas the Yurith sub-catchment, with higher elevation and greater soil coverage from forests, woodlands, tea plantations and small-holder farms (Masese et al., 2012). Stenfert Kroese et al. (2020b) assessed seasonal sediment variation in three contrasting catchments in the Yurith sub-catchment. Over four years, they found that subsistence agricultural practices contributed significantly more sediment compared to tea and tree plantations and natural forests. Moreover, their research highlighted that natural forests, and tea or tree plantations delivered water to streams through subsurface pathways, whereas surface runoff was dominant in agricultural catchments.

The Awach sediment core exhibited a gradual divergence of sediment contributions (Supplementary Table 24) between the Lower Awach sub-catchment (Kibuon, Kasipul, Owade sub-catchments) and the Upper Awach sub-catchment (Kibondo sub-catchment), with the Lower Awach sub-catchment gradually decreasing proportionally as the lowest overall contribution from 1960 to 2005. The Lower Awach sub-catchment 1 inputs increased from ~ 2006 to 2012, briefly and only slightly greater than the Upper Awach sub-catchment, as the main contributor to the overall peak in sedimentation rate during this period (0.3–0.5 g cm^2^ yr^−1^). Overall, the sedimentation rate for the Awach core increased steadily from the 1960s and then a peak increasing from spanning the early 2000s coinciding with the relative increase in contributions from the Lower Awach sub-catchment. Changes within the Lower Awach sub-catchment resulted from a conversion to moderately vegetated cover in the lower regions of the sub-catchment (Kibuon) due to the development of roads, settlements and farmland and from timber harvesting from forest farms and natural forests for timber and fuel in the upper reaches of the Lower Awach sub-catchment (Owade/Kasipul). A later peak in sedimentation at up to 0.9 g cm^2^ yr^−1^ between 2010 and 2020 coincided with the relative increase in sediment contribution from the Upper Awach sub-catchment, which experienced similar pressures from forest farms for timber and fuel and conversion to agriculture, exacerbated by the topographic challenges of steep slopes and higher rainfall compared to the lower reaches of the Awach basin. Hellens et al. (2024) reported a comparative increase in sediment yield in the Upper Awach sub-catchment of 23% and in the three sub-catchments that make up the Lower Awach sub-catchment, a 17–33% increase between 2018 and 2023.

### Implications for management of findings

The combination of sediment geochronology and source apportionment provides a powerful framework for understanding how land-use change influences long-term land-to-lake transfers within the Lake Victoria Basin. By linking temporal trends in sediment accumulation with changing sediment provenance, this study demonstrates that accelerated sedimentation in the Winam Gulf is not solely the result of diffuse catchment-wide degradation but is increasingly associated with specific sub-catchments undergoing forest clearance, agricultural expansion and urban development. The marked increases in sedimentation rates observed in the Nyando and Sondu-Miriu catchments since the 1960s, and particularly after 2000, indicate a substantial intensification of erosion processes and sediment connectivity across the landscape.

From an environmental geochemistry perspective, increased sediment delivery represents more than the physical transfer of soil. Eroded sediments act as vectors for the transport of organic matter, nutrients, carbon and other geochemical constituents from terrestrial environments into aquatic ecosystems. Temporal increases in phosphorus, sulphur, calcium and organic matter observed in several sediment cores suggest increasing mobilisation of agriculturally influenced surface soils over recent decades. Such transfers can contribute to eutrophication, altered nutrient cycling and declining aquatic ecosystem health within the Winam Gulf, reinforcing concerns previously raised regarding catchment-derived pressures on Lake Victoria.

The impacts are also felt within terrestrial systems. The erosion of fine-grained, nutrient-rich topsoil reduces soil fertility, organic carbon storage and agricultural productivity, thereby increasing pressure on already vulnerable farming systems. The substantial increases in sedimentation rates observed across all major river inputs therefore reflect a dual loss: degradation of productive soil resources upstream and an increasing sediment and nutrient burden on downstream waters. This linkage highlights the importance of viewing soil health, water quality and food security as interconnected components of a single land-lake system rather than as separate management challenges.

The source apportionment results provide practical opportunities for targeted intervention. Rather than applying mitigation measures uniformly across entire catchments, management efforts can be directed towards the sub-catchments identified as disproportionate contributors to sediment delivery during periods of accelerated erosion. Measures such as protection of remaining forest cover, agroforestry, terracing, cover cropping, riparian buffer restoration and improved management of cultivated slopes are likely to provide the greatest benefit when implemented within these priority source areas. Combining the multi-decadal perspective provided by sediment cores with seasonal monitoring, Earth observation data and process-based erosion models would further improve the targeting and evaluation of mitigation strategies.

Importantly, the sediment record suggests that concerns regarding accelerated land degradation within the Lake Victoria Basin remain unresolved. The call for action by the World Agroforestry Centre (WAC 2006) who warned of a future scenario of accelerated land degradation from poor land management would appear to have been unheeded, despite extensive efforts in community engagement and capacity building at the time. Stakeholder consultations undertaken during this project identified fragmentation of governance, monitoring activities and environmental data as persistent barriers to effective management (Aura et al., 2025; Humphrey et al., 2023, 2024). Greater integration between agricultural, forestry, water-resource and fisheries sectors at catchment scale will therefore be required to translate scientific evidence into effective land-lake management strategies (Njagi et al., 2022; Hellens et al., 2024; Humphrey et al., 2025; Wynants et al., 2020).

Ultimately, the value of the integrated approach presented here lies in its ability to identify where sediments originate, how contributions have changed through time and which areas should be prioritised for intervention. The valuation of perceived provisioning services of a forest landscape is needed to justify protection upstream (Miller et al., 2021), alongside the cost of non-intervention, estimated to be USD 390 million per annum (Cohen et al., 2006) to Kenya’s economy from soil erosion alone. However, the culture of staple traditional crops such as maize which typically occurs during the heavy rainy season adversely exposes agricultural farm-to-soil erosion (Kroese Stenfert et al. 2020a; Humphrey et al., 2022). Such an integrated approach is essential for protecting soil resources, reducing nutrient and sediment transfer to Lake Victoria, improving ecosystem resilience and supporting the long-term food, livelihood, environmental security and health (Watts et al., 2019, 2021) of communities dependent on the basin.

## Conclusion and recommendations

This study demonstrated a substantial acceleration in sediment delivery from the major river catchments of the Winam Gulf since the 1960s, with the most rapid increases occurring after 2000. Sedimentation rates increased by approximately 700% in the Nyando catchment and 300% in the Sondu-Miriu catchment since the 1960s, coinciding with widespread land-use change, forest loss, agricultural expansion and urban development across the Lake Victoria Basin. Temporal trends in sediment geochemistry indicated increasing mobilisation of surface soils and associated nutrients, while source apportionment analysis revealed that changes in sedimentation were linked to shifts in the relative importance of specific sub-catchments rather than uniform changes across entire basins.

The principal contribution of this study is the integration of Pb-210 geochronology with sediment-source fingerprinting to provide a multi-decadal reconstruction of land-to-lake transfers. This approach enabled both the timing and the origin of changing sediment inputs to be identified, providing a stronger evidence base for catchment management than either method alone. Linking historical sediment records with sediment provenance offers a practical means of identifying priority source areas and evaluating the long-term consequences of land-use change on sediment delivery.

From an environmental geochemistry and health perspective, accelerated erosion represents the transfer of nutrient-rich topsoil, organic carbon and associated geochemical constituents from productive agricultural landscapes into aquatic ecosystems. These land-lake transfers have implications for soil fertility, water quality, eutrophication, fisheries productivity and food security throughout the basin. The results therefore highlight the need to manage catchments as integrated land-lake systems in which terrestrial and aquatic environmental health are intrinsically linked.

The framework developed here provides a basis for targeting mitigation measures within critical source areas, supporting more efficient deployment of limited resources and improving the evaluation of restoration outcomes. Combined with Earth Observation, seasonal monitoring and stakeholder-led management approaches, this methodology has considerable potential to support adaptive catchment management in the Lake Victoria Basin and other rapidly developing catchments facing similar challenges worldwide. Ultimately, reducing soil erosion is not simply a matter of limiting sediment loss; it is essential to protect soil resources, ecosystem services, water quality and the resilience of communities that depend upon them.

## Supplementary Information

Below is the link to the electronic supplementary material.

Supplementary file1 (XLSX 1342 KB)

## Data Availability

All raw data is provided in the supplementary information associated with this article for full transparency and use by other researchers.
